# Ultrasound-guided lung biopsy with coaxial technique: pleural contact length affects the occurrence of pneumothorax after first puncture

**DOI:** 10.1007/s11604-021-01213-6

**Published:** 2021-11-05

**Authors:** Rinpei Imamine, Takeshi Kubo, Keizo Akuta, Hisato Kobayashi, Yoshiharu Yamamoto, Ayako Saito, Naoki Sakai, Tomoyuki Shirase

**Affiliations:** 1 Department of Diagnostic Radiology, Kurashiki Central Hospital, 1-1-1 Miwa, Kurashiki, Okayama 710-8602 Japan; 2 Department of Radiology, Japanese Red Cross Otsu Hospital, 1-1-35, Nagara, Otsu, Shiga 520-8511 Japan; 3 Department of Diagnostic Imaging and Nuclear Medicine, Kyoto University Graduate School of Medicine, 54-Kawaharacho, Shogoin, Sakyoku, Kyoto, 606-8507 Japan; 4 Department of Radiology, Tenri Hospital, 200 Mishima, Tenri, Nara 632-8552 Japan; 5 Department of Radiology, Kobe Asahi Hospital, 3-5-25, Boojicho, Nagataku, Kobe, Hyogo 653-0801 Japan; 6 Kurashiki Clinical Research Institute (Ohara Health Care Foundation), Kurashiki Central Hospital, 1-1-1 Miwa, Kurashiki, Okayama 710-8602 Japan; 7 Department of Respiratory Medicine, Japanese Red Cross Otsu Hospital, 1-1-35, Nagara, Otsu, Shiga 520-8511 Japan; 8 Department of Pathology, Japanese Red Cross Otsu Hospital, 1-1-35, Nagara, Otsu, Shiga 520-8511 Japan

**Keywords:** Ultrasound, Percutaneous lung biopsy, Coaxial technique, Pneumothorax after first puncture, Pleural contact

## Abstract

**Purpose:**

To assess prebiopsy characteristics influencing the occurrence of pneumothorax after first puncture of ultrasound (US)-guided lung biopsy with coaxial technique.

**Materials and methods:**

From January 2007 to September 2018, 180 peripheral lung lesions in 174 patients who underwent B-mode US-guided lung biopsy with coaxial technique at single institution were included in this study. Technical success was defined as the ability to make a diagnosis using the acquired sample with/without an adverse event of pneumothorax. Statistical analyses of prebiopsy characteristics were performed to identify the most important cutpoint and to evaluate the effect on diagnostic accuracy.

**Results:**

Of the 180 lesions (mean size, 37 mm ± 26.2; mean pleural contact length, 38.2 mm ± 34.4), technical success rate was 97.2% (175/180 lesions) and diagnostic accuracy rate was 91.6% (165/180 lesions). Pneumothorax occurred immediately after first puncture for seven of 180 lesions. Classification and regression tree analysis and Fisher’s exact test showed the proportion of the pneumothorax immediately after first puncture was higher in lesions with pleural contact length less than 9.78 mm (*p* = 0.002). No significant difference was shown between the pneumothorax and non-pneumothorax after first puncture in technical success and final diagnosis success rate.

**Conclusion:**

Pleural contact length affects the occurrence of pneumothorax after first puncture of US-guided lung biopsy with coaxial technique.

## Introduction

Ultrasound (US)-guided transthoracic biopsy of peripheral pulmonary masses was introduced in 1976 [1]. Regarding parenchymal lung lesions adjacent to and/or abutting the pleura, percutaneous biopsy can be performed under both computed tomography (CT) and US guidance. Although several studies have described US guidance to be superior to CT guidance [2–5], US-guided lung biopsy is not yet fully performed. One of the reasons may be technical difficulty because US images are strongly affected by the air in the lungs. An US-guided lung biopsy cannot be performed when the target lung lesion becomes invisible due to a small amount of pneumothorax during sampling.

In the previous reports, the diagnostic accuracy of US-guided transthoracic lung biopsy ranged from 76 to 97% [2–8]. As a predictive factor for diagnostic accuracy of this procedure, size and pleural contact length were reported [3, 5, 7, 9]. Bleeding and pneumothorax are the most common complications of percutaneous US-guided lung biopsy. The incidence of hemoptysis ranged from 0.5 to 22%, and hemothorax is rare [2, 3, 5, 6, 9–11]. Air bronchial sign was reported as a risk factor of hemoptysis [11]. The pneumothorax incidence rate ranged from 0 to 5.8%, while the rate of chest tube drainage ranged from 0 to 1% [3, 4, 12]. Pneumothorax after the first attempt of tissue sampling might adversely affect the outcome of the image-guided biopsy because the air in the pleural space would prevent further US-guided puncture. To the best of our knowledge, there has been no study of the risk factor of pneumothorax related to US-guided puncture. We hypothesized that the risk of air leak might be inversely related to the area of the pleura abutting the target lesion. Acknowledgement of the risk of pneumothorax at the first puncture would be helpful for operators to choose the most appropriate method of image-guided biopsy among US-guided, CT-guided, or hybrid techniques. The purpose of this study was to assess prebiopsy characteristics influencing the occurrence of pneumothorax after first puncture of ultrasound (US)-guided lung biopsy with coaxial technique.

## Materials and methods

The institutional review board approved this retrospective single-center study and waived the requirement for informed consent.

### Patients

**Fig. 1:**
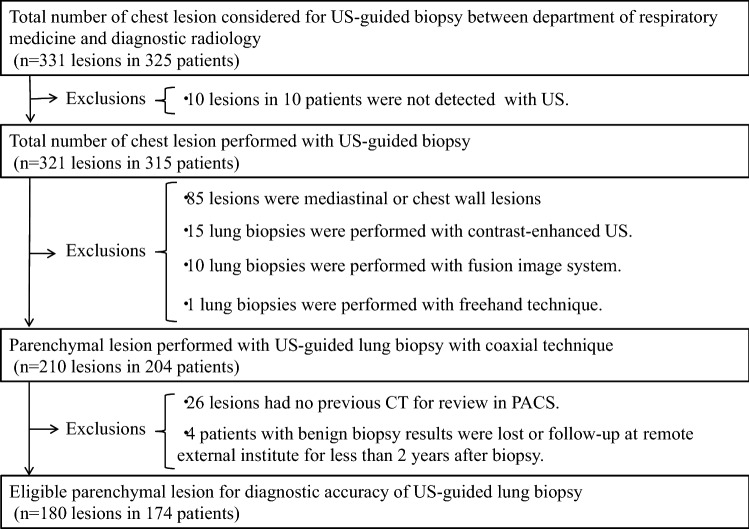
Flowchart of lung lesion performed with US-guided percutaneous biopsy in this study. Flowchart of US-guided lung biopsy demonstrates the overall study and exclusion criteria for this study. *US* ultrasound, *PACS* Picture Archiving and Communications Systems

From January 2007 to September 2018, 325 consecutive patients were considered for US-guided chest biopsy between the department of respiratory medicine and diagnostic radiology. Ten lesions in ten patients were not detected by US, though all patients underwent US to detect the potential biopsy site of chest lesions by an interventional radiologist (H.K.; with experience of more than 30 years). The remaining 315 patients who underwent US-guided chest biopsies were identified by reviewing the center’s electronic medical records. The review revealed 321 chest lesions of 315 patients, six of whom underwent US-guided biopsies for two different lung lesions on separate occasions. A biopsy was performed for all patients within four weeks after a chest lesion was discovered using CT. From these candidates, 141 lesions in 141 patients were judged to be ineligible based on the exclusion criteria and therefore were excluded from further analysis. The remaining 180 peripheral lung lesions, defined as parenchymal lesions adjacent to or abutting the pleura with CT, in 174 patients were examined in this study (Fig. 1).

### US-guided lung biopsy procedures

All procedures were a coaxial technique performed by the same interventional radiologist (H.K.; with experience of more than 30 years) assisted with fellowship-trained radiologists. The interventional radiologist determined the needle guide line using both a high-frequency linear probe and a low-frequency microconvex probe. The GE LOGIQ Q9 US imaging system (GE Healthcare, Milwaukee, Wisconsin, USA) with a linear probe (4–9 MHz) and a microconvex probe (2–5 MHz) or the Xario SSA-660A/LG US imaging system (Toshiba, Tokyo, Japan) with a linear probe (6–11 MHz) and a microconvex probe (3–6 MHz) were used. For all biopsies during the procedure, the interventional radiologist maintained probe position and an 18-gauge introducer needle (US-guided puncture needle; Create Medic Co., Ltd, Yokohama, Japan), which was inserted by needle guidance into the chest wall near the contact area between the lesions and pleura under local anesthesia using 1% lidocaine (Xylocaine; Astra-Zeneca, Osaka, Japan). If the lesion diameter was < 20 mm, the tips of the needles were placed at least 20 mm away from the posterior margin of the lesion–lung interface. Moreover, a fellowship-trained radiologist prepared two 20-gauge automatic cutting needles with a throw length of 20 mm (Bard Magnum; Bard Biopsy Systems, Tempe, AZ, USA). After first biopsy needle was introduced through the introducer needle, the patients were instructed to hold their breath at the optimal phase of respiration so that the tip of the needle accurately penetrated the lesion through the pleural contact area. During a breath-hold without a break, first and second biopsy needles were punctured and withdrawn sequentially by a fellowship-trained radiologist. The patient exhaled after the interventional radiologist withdrew the introducer needle from the body. Only one sample was obtained if the target lung lesion disappeared due to pneumothorax immediately after the first puncture. On exposure of the introducer needle to room air during withdrawal of the biopsy needle, the interventional radiologist closed the hole made by the introducer needle with a finger to prevent air from flowing into the lung or the pleural cavity. When the operators considered the quantity of the specimen insufficient after two punctures through visual examination, the procedure was repeated. All patients were admitted for observation overnight after the procedure. If the patients complained of symptoms, a chest CT was obtained. After obtaining a posteroanterior chest radiograph, the uncomplicated patients were discharged.

### Assessment of the lesion characteristics

**Fig. 2:**
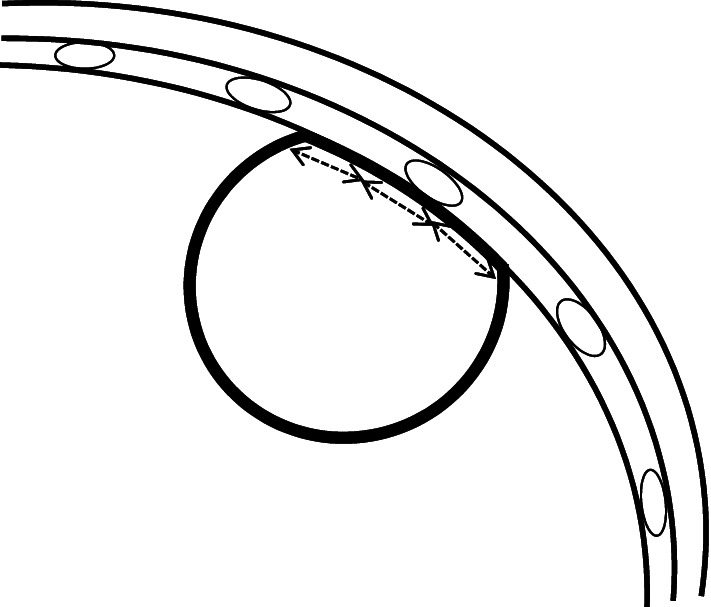
Schematic view to measure the pleural contact length. The pleural contact length was approximated by a broken line consisting of straight lines of 10 mm or less on the mediastinal window of the CT image. The maximum contact length was defined as the length of the line of contact between the lesion and the chest wall in the maximum cross section of the lesion

Parenchymal lung lesion adjacent to and/or abutting the pleura was defined as any area of pulmonary opacification surrounded by normal pulmonary parenchyma and visceral pleura on the CT image. No ground grass opacity lesions were found in this study. Two authors (R.I. and K.A.) who did not participate in any biopsy procedures in this study independently measured the longest diameter and pleural contact length of all lesions from the CT images with a PACS viewer (RadiForce GX320, EIZO, Japan). The longest diameter was measured along the long axis of the lesion in the mediastinal window setting of the CT image. The maximum contact length was defined as the length of the line of contact between the lesion and the chest wall in the maximum cross section of the lesion on the mediastinal window of the CT image. Since this line is curved, it was approximated by a broken line consisting of straight lines of 10 mm or less and the total length of all the straight lines was used as the maximum contact length (Fig. 2). The pleural lesion contact area was defined as the area of the peripheral lung lesion in contact with the overlying pleural surface on the CT mediastinal section. Mean values were accepted for all quantitative data.

### Appraisal of the procedure results

The pathological results of the biopsy specimens were classified as malignant, benign, and inconclusive-diagnostic due to inadequate tissue. Clinical diagnosis of the malignant tumor was made on the histopathological diagnosis of malignancy. Clinical diagnosis of benign was confirmed pathologically on the surgical specimen or clinically with the subsequent observation of the lesion showing the disappearance or no increase in the size of the lesion on follow-up CT for at least 2 years. The final diagnosis was defined as a clinical diagnosis as of March 2019. The diagnostic accuracy of US-guided biopsy is defined as the number of accurately diagnosed lesions divided by the total number of lesions. Technical success was defined as the ability to make a diagnosis using the acquired sample with/without an adverse event of pneumothorax.

### Statistical analysis

Data analyses were performed with SPSS software (version 25.0 for Windows; IBM Corp., Armonk, NY, USA). Pearson coefficient was calculated for correlations. Classification and regression trees (CART) and Fisher’s exact test were used to find the importance of variables affecting the occurrence of pneumothorax after the first puncture. The classifications trees were elaborated using the Gini splitting rule to maximize the homogeneity of child nodes with respect to the value of the dependent variable. It reaches it minimum (zero) when all cases in a node fall into a single category. The improvement value is shown for independent variable statistics of CART. Minimum change in improvement is the minimum decrease in impurity required to split a node. A node is not split if impurity would decrease less than 0.0001. Fisher’s exact test was used to evaluate the effect of the most important variable in technical success and final diagnosis success rate. Statistical significance was determined at *p* < 0.05.

## Results

The patients’ demographics, lesion characteristics, and US equipment for biopsy are summarized in Table 1. The biopsy results of 180 lesions (mean size, 37 ± 26.2 mm; mean pleural contact length, 38.2 ± 34.4 mm) were 138 malignant lesions, 37 benign lesions, and 5 inconclusive diagnostic lesions due to inadequate tissue. Therefore, the technical success rate was 97.2% (175/180 lesions). Of 5 inconclusive diagnostic lesions, pneumothorax occurred immediately after the first puncture in one lesion. The correct diagnoses with biopsy were made for 165 lesions (138 true-malignant lesions and 27 true-benign lesions); thus, diagnostic accuracy was 91.6% (165/180 lesions). Final diagnoses of histopathological characteristics for all lesions are summarized in Table 2. Fifteen diagnostic failure cases are summarized in Table 3. Five inconclusive diagnostic lesions were finally diagnosed as 4 lesions of primary lung cancer and one benign lesion, which were confirmed by lobectomy (No.1, No.2 and No.3) and US-guided re-biopsy for lung lesion (No.4 and No.5). Of patients who obtained benign biopsy results, 10 lesions in 10 patients were confirmed as malignant by lobectomy (No.6, No.7, No.8, No.9, No.12 and No.15), trans-bronchial lung biopsy (No.10), US-guided biopsy for another lung lesion (No.11), US-guided biopsy for subclavicular lymph node (No.14) and clinical malignant course (No.13). Patient with clinical malignant course was suspected of recurrence of squamous lung cancer and thereby died at 591 days after procedure.

**Table 1:** Patients’ demographics, lesion characteristics and US equipment for biopsy

	Value
Mean age(years)	71.8 ± 9.6
Sex	
Female	45
Male	135
Size (mm)	37 ± 26.2
Pleural contact length (mm)	38.2 ± 34.4
Lesion location	
Right upper lobe	36
Right middle lobe	4
Right lower lobe	58
Left upper lobe	46
Left lower lobe	36
US equipment	
GE	136
TOSHIBA	44

**Table 2:** Final diagnosis of histopathological characteristics

Final diagnosis	Value	Diagnostic accuracy
Malignant lesion (*n* = 153)		
Adenocarcinoma	72	90.3% (65/72)
Squamous cell carcinoma	36	91.6% (33/36)
Small cell lung carcinoma	17	94.1% (16/17)
Mucoepidermoid carcinoma	1	100% (1/1)
Spindle cell carcinoma	1	100% (1/1)
Large cell neuroendocrine carcinoma	1	100% (1/1)
Combined large cell neuroendocrine carcinoma	1	0% (0/1)
Undifferentiated carcinoma	3	100% (3/3)
Metastasis from extrathoracic organs	16	93.8% (15/16)
Solitary fibrous tumor	3	100% (3/3)
Malignant mesothelioma	1	100% (1/1)
Malignant lymphoma	1	100% (1/1)
Benign lesion (*n* = 27)		
Organized pneumonia	5	100% (5/5)
Hamartoma	1	100% (1/1)
Aspergilloma	1	100% (1/1)
Nocardiosis	1	100% (1/1)
Round atelectasis	1	100% (1/1)
Epithelioid cell granuloma	2	100% (2/2)
IgG4-related disease	1	100% (1/1)
Non-specific inflammation and fibrosis	15	93.3% (14/15)

**Table 3:** Diagnostic failure cases

No	Size	PCL	Pnx	Biopsy result	Final diagnosis
Histological inconclusive diagnostic biopsy result					
1	10.5	11	−	Atypical cell	Adenocarcinoma(Invasive mucinous)
2	21	22	+	Adipose fibrous tissue	Adenocarcinoma
3	34.8	29.6	−	Atypical epithelial cells	Inflammation and fibrosis
4	41	26.4	−	Nectoric tissue	Adenocarcinoma
5	117	129	−	Nectoric tissue	Squamous cell carcinoma
Histological benign biopsy result					
6	9.8	11.2	−	Fibrous tissue	Squamous cell carcinoma
7	14.3	12.2	−	Atypical granular cell	Combined large cell neuroendocrine carcinoma
8	15.5	11.3	−	Chronic pneumonitis	Small cell carcinoma
9	16.5	14.5	−	Fibrous tissue	Adenocarcinoma(Invasive mucinous)
10	20.5	27.6	−	Xanthogranuloma	Adenocarcinoma(Invasive mucinous)
11	20.5	27.5	−	Inflammation and fibrosis	Adenocarcinoma
12	25	15	−	Fibrous tissue	Metastasis of mucinous cystadenocarcinoma
13	28.5	12.4	−	Fibrous tissue	Squamous cell carcinoma
14	31	44	−	Lymphoid infiltration	Adenocarcinoma
15	68.4	74.4	−	Inflammation	Mucoepidermoid carcinoma

Size and PCL of lung lesions are shown in millimeters

*PCL* pleural contact length, *Pnx* pneumothorax after first puncture

At least one uneventful puncture(s) without pneumothorax were completed for 173 lesions, whereas pneumothorax occurred immediately after the first puncture in seven of 180 lesions (3.9%). In addition, all complications were included as follows: asymptomatic pneumothorax (5%; 9/180), self-limited hemoptysis (3.8%; 7/180), and empyema (0.6%; 1/180). No fatal complications were observed. All nine patients with pneumothorax recovered spontaneously without placement of a chest tube. Hemoptysis in seven patients occurred after procedures and resolved spontaneously with hemostatic agents. One patient developed empyema two days after discharge and resolved for one week with antibiotics.

**Fig. 3:**
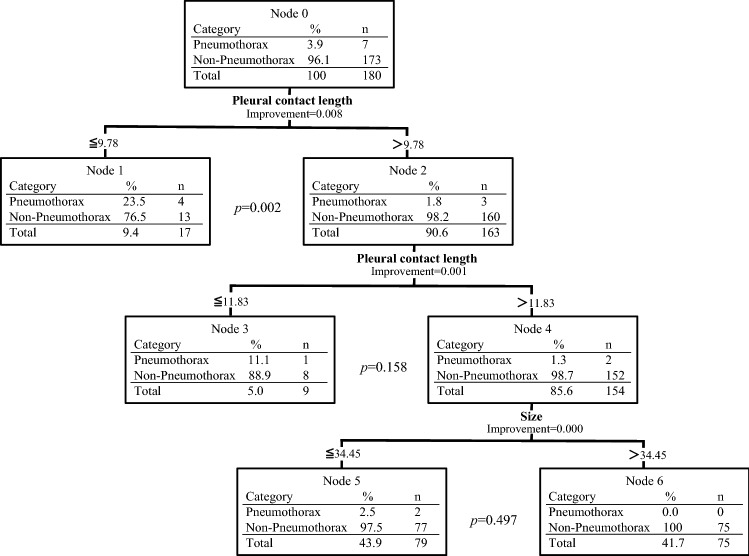
The assessment of prebiopsy characteristics for the occurrence of pneumothorax after first puncture using classification and regression tree analysis and Fisher’s exact test. Classification and regression tree analysis and Fisher’s exact test showed that the rate of pneumothorax incidence after first puncture was higher in lesions with pleural contact length less than 9.78 mm (*p* = 0.002). The classifications trees were elaborated using the Gini splitting rule to maximize the homogeneity of child nodes with respect to the value of the dependent variable. The improvement value is shown for independent variable statistics

Variables were included as follows: age, sex, US equipment, lesion location, pleural contact length and size. A positive correlation between the pleural contact length and the size is observed (*r* = 0.81), but correlation between other values is not detected. In terms of the prebiopsy characteristics for the occurrence of pneumothorax after first puncture, CART and Fisher’ exact test showed that the rate of pneumothorax incidence after first puncture was higher in lesions with pleural contact length less than 9.78 mm (*p* = 0.002). The result was presented in Fig. 3. Four (23.5%) of 17 lesions with pleural contact length less than this cut-off occurred pneumothorax immediately after first puncture, whereas 3 (1.8%) of 163 lesions larger than this cut-off occurred pneumothorax immediately after first puncture. The 163 lesions were then divided according to a pleural contact length threshold of 11.83 mm and a size threshold of 34.45 mm. Fisher’ exact test showed no significant differences in the proportion of lesions that occurred pneumothorax immediately after first puncture at these levels of the classification tree.

Of the seven lesions that disappeared due to pneumothorax after the first puncture, tissue was adequate for pathological analysis and the final diagnoses were correct with biopsy results in sex lesions. There was no statistically significant difference between the pneumothorax and non-pneumothorax groups after the first puncture in technical success and final diagnosis success rates. The results are summarized in Table 4.

**Table 4:** Comparison of pneumothorax group and non-pneumothorax group after first puncture in technical success and final diagnosis success rate

	Pneumothorax after first puncture (*n* = 7)	Non-pneumothorax after first puncture (*n* = 173)	*p* value
Technical success, *n* (%)	6/7 (85.7%)	169/173 (97.7%)	0.182
Final diagnosis success, *n* (%)	6/7 (85.7%)	159/173 (91.9%)	0.462

## Discussion

We demonstrated that the technical success rate was 97.2% and the diagnostic accuracy was 91.6% for US-guided lung biopsy with coaxial technique for parenchymal lung lesions adjacent to and/or abutting the pleura without life-threatening complications. Seven of 180 lesions (3.9%) became invisible due to pneumothorax immediately after the first puncture. CART and Fisher’s exact test showed the proportion of the pneumothorax immediately after the first puncture was higher in lesions with pleural contact length less than 9.78 mm (*p* = 0.002). No significant difference was shown between the pneumothorax and non-pneumothorax groups after the first puncture in technical success and final diagnosis success rate. In all procedures of this study, puncture line and puncture timing were decided by the same interventional radiologist; therefore, prebiopsy characteristic affects the pneumothorax incidence immediately after the first puncture.

Seven lesions became invisible due to pneumothorax immediately after the first puncture. Seven cases with pneumothorax might have had the erroneously identified pleural contact area punctured at the first attempt. A false-positive pleural contact area may be observed by a linear and micro convex probe, even if the lesion conspicuity is considered sufficient for puncture. The correct detection of pleural contact area needs to be attainable with careful US examination. However, there are some causes of false-positive identification of pleural contact areas as pleural adhesions [13, 14]. Tasci et al. [15] described the linear probe is superior to the sector probe for identifying pleural pathologies in superficial area. A linear probe has higher resolution of image than a microconvex, whereas a microconvex probe has the advantage of being a useful tool through the intercostal space due to a narrow width of probe. With respect to successful sampling of peripheral small lung lesions with US guidance, US image is important to avoid the pleura injury by the needle tip. Novel methods to evaluate lung sliding using Doppler or M-mode images may help avoid puncturing aerated parenchyma [16, 17].

CART shows short pleural contact length is related to the occurrence of pneumothorax after first puncture. Generally, it is difficult to make a needle tip traverse accurately the narrow pleural contact area in an optimal respiratory phase. In this study, air leakage from the normal lung made by first puncture must be the leading cause of pneumothorax, because the hole of the introducer needle was closed with a finger to prevent air from flowing into the lung or the pleural cavity. As described above, pneumothorax can occur when the needle penetrates normal visceral pleura and lung tissue outside the lesion. Although there have been no previous reports of the pneumothorax incidence related to US-guided puncture, the incidence rate of asymptomatic pneumothorax immediately after first puncture may reflect the potential risk of puncturing normal visceral pleura and lung parenchyma surrounding target lung lesions. Therefore, it seems reasonable to employ pleural contact length as a marker for technical difficulty of US-guided lung biopsy.

The present study did not find statistically significant differences between the pneumothorax and non-pneumothorax groups after the first puncture in technical success and final diagnosis success rate. Regarding factors affecting diagnostic failure of US-guided lung biopsy, Guo et al. [6] described the technical difficulty associated with small lesion and respiratory movement, insufficient specimens associated with complications (pneumothorax and bleeding), and the presence of necrosis in the lesion. In this study, biopsy results were necrotic tissue in 2 of 15 diagnostic failure lesions. However, the observation in our study is inconsistent with the report that pneumothorax leads to inadequate tissue for histopathology examination due to impaired sampling. The reason for this contradiction remains somewhat unclear, but it may be the effect of the breath-hold during sequential punctures. Although there are some reviews about a variety of US-guided lung biopsy techniques [18], the acquisition of two specimens during single breath-hold were not reported. Given the cellularity requirement for molecular testing, a single pass using a 20-gauge side-cut device may not be enough, even if the target volume was fulfilled (19). The unique technique without radiation in this study showed the potential to be a feasible and reliable biopsy technique in the era of personalized medicine in oncology.

This study had some limitations. First, the study is a retrospective single-arm study that used samples performed by a single interventional radiologist as an operator in a single center. Our study was not designed to compare prebiopsy characteristics between expert radiologists and fellows; therefore the effect of operators’ experience of the procedure cannot be determined. Second, there may have been selection bias, because only thoracic lesions that could be visualized with US were selected. Third, unexpected events during sampling as a reason for pneumothorax were not evaluated in detail. There may have been some cases of error between the needle guideline and the actual needle path or breath-hold failure. Finally, the relationship between the US probe or the patients’ respiratory function and the occurrence of pneumothorax after the first puncture was not evaluated. The influence of these factors was not elucidated, and a dedicated study is desirable.

In conclusion, pleural contact length affects the occurrence of pneumothorax after the first puncture of US-guided lung biopsy with coaxial technique.
